# Primary health care in China: A decade of development after the 2009 health care reform

**DOI:** 10.1002/hcs2.14

**Published:** 2022-09-12

**Authors:** You Wu, Zeyu Zhang, Ning Zhao, Yue Yan, Lina Zhao, Qi Song, Rong Ma, Changfeng Li, Jinyi Li, Suibin Liu, Xinran Bi, Zongjiu Zhang

**Affiliations:** ^1^ School of Medicine Tsinghua University Beijing China; ^2^ Institute for Hospital Management Tsinghua University Beijing China

**Keywords:** policy, primary health care, public health service, universal health coverage

## Abstract

Over 40 years ago, primary health care (PHC) was defined in the Alma‐Ata Declaration as a critical component of the health care system to address the basic health demand of the people. In China, the Government attaches great importance to health care at the primary level. After the launch of the historical Reform of the Medical and Health Care System in 2009, the PHC system in China has witnessed major progress and breakthroughs, especially in its steadily increased capacity, continuously improved accessibility, and betterment in equality. In this review, we summarized published literatures and official policies, synthesized data from the electronic registration information system of the National Health Commission, national statistical reports, and yearbooks in health care. The review is intended to describe the systematic development of PHC in China in the last decade. The main results include: the solid national policy foundation, increasing number of PHC institutions and workforce, better training of PHC professionals, major achievements in primary health indicators, government financial support to PHC institutions, improved PHC budgeting and insurance coverage, and the advancement of supporting technologies. Challenges and prospects are also discussed.

## Graphical abstract

After the launch of the historical Reform of the Medical and Health Care System in 2009, China's primary health care (PHC) system has witnessed significant progress and breakthroughs. In this review, we summarized published literature and official policies and synthesized data from the electronic registration information system of the National Health Commission, national statistical reports, or yearbooks in health care. The review describes the structural design and major achievements of PHC in China in the last decade, emphasizing the national policy foundation, budgeting and insurance coverage, evolution of service delivery, professional team development, and the advancement of supporting technologies.

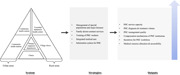

**Abbreviations**
CDCs: Centers for Disease Control and Prevention
CNY: Chinese yuan Renminbi
PHC: primary health care
SARS: severe acute respiratory syndrome
TCM: traditional Chinese medicine
WHO: World Health Organization

## 1 INTRODUCTION

In 1948, the World Health Organization (WHO) mentioned in its Charter that primary health care (PHC) should focus on maternal and child health and epidemic prevention [1]. In 1978, in the Declaration of Alma‐Ata, WHO introduced the concept of “health for all by the year 2000” as a goal to enhance equity in global health services [2]. According to the Declaration, PHC should focus on addressing the main health problems of the population, including health promotion, disease prevention, rational treatment, and community‐based rehabilitation. In 2018, the Astana Declaration further refined the concept of PHC, emphasizing the importance of a personal health gatekeeper system with integrated health services characterized by: (1) life‐course care (from health promotion, prevention, treatment, rehabilitation to palliative care); (2) continuity and sustainability; and (3) high quality and efficiency [3].

In China, the Government attaches great importance to health care at the primary level. In recent years, China's PHC system has been evolving constantly under the strong leadership and organization, accompanied by the concerted efforts of the vast number of health care workers. The system has witnessed major progress and breakthroughs, especially in its steadily increased capacity, continuously improved accessibility, and betterment in equality [4, 5].

In this review, we aim to describe the structural design, systematic development, and major achievements of PHC in China after the historical Reform of the Medical and Health Care System in 2009 [6, 7]. It emphasizes the national policy foundation, budgeting and insurance coverage, evolution of service delivery, professional team development, and the advancement of supporting technologies. We composed the narrative review based on published literatures and official policies; all relevant data were accessed and summarized from the electronic registration information system of the National Health Commission [8], national statistical reports [9, 10], or yearbooks in health care [11, 12, 13]. Opinions from experts and key stakeholders were also collected to generate suggestions and recommendations for future improvement of PHC in China.

## 2 POLICY FOUNDATION

### 2.1 Before 2009

Following the establishment of the People's Republic of China in 1949, a nationwide health and epidemic prevention system was established from the ground up. The early goal of health care in China was to improve urban and rural sanitation through the patriotic health campaigns. After the severe acute respiratory syndrome epidemic (SARS) in 2003, the nation's public health initiatives have gotten renewed focus. Thereafter, the system has been built around disease prevention and control, with relevant laws and regulations being enacted one after another. However, there is still tremendous opportunity for improvement in the design of the basic health care service delivery systems.

*The Opinions of the State Council on the Development of Urban Community Health Services* advocated in 2006 to “focus on encouraging system and mechanism innovation to provide residents with safe, effective, convenient, and affordable public health services and basic medical services” [14]. In the same year, the *Administrative Measures for Urban Community Health Service Institutions (Trial Version)* proposed 12 public health service items that community health service institutions should provide [15]. The 12 services include (1) health information management; (2) health education; (3) prevention and control of infectious diseases, endemic diseases, and parasitic diseases; (4) chronic disease prevention and control; (5) mental health services; (6) women's health care; (7) children's health care; (8) elderly health care; (9) disability rehabilitation guidance and training; (10) family planning technical advice and guidance; (11) assistance in response to public health emergencies within the jurisdiction; and (12) other relevant services. These categories have become the framework for basic public health services in China.

### 2.2 After 2009

In 2009, the *Opinions on Deepening the Reform of the Medical and Health System* issued by the Central Committee of the Communist Party of China and the State Council provided a policy basis for the construction of the public health service system [7, 16]. This policy sets “promoting equal access to basic public health services” as one of the five key components of the reform.

In 2012, the *Plan for Deepening the Reform of the Medical and Health Care System during the Twelfth 5‐Year Plan Period* proposed to “provide the basic medical and health care system as a public product to the people” and to “promote the equalization of basic public services” [17]. In the same year, the former Ministry of Health put into effect the *National standards for basic public health services* [18] and the *Opinions on Strengthening the Performance Evaluation of Basic Public Health Service Projects* [19].

In 2016, the “Healthy China 2030” initiative was launched with four guiding principles: (1) prioritizing health; (2) reform and innovation; (3) scientific development; and (4) fairness and justice. The policy emphasizes the importance of “focusing on rural areas, promoting the equalization of basic public health services, and maintaining the public welfare of basic medical and health services.” These actions have gradually reduced disparities in basic health services between urban and rural areas, paving the way for universal health coverage and social equity [20].

In 2017, a number of service items were added in addition to the previous two editions of the National Basic Public Health Service program [21]. The amendment demonstrated that the scope and coverage of basic public health service are flexibly adjusted in response to the advancement of the health care reform and the improvement of people's health status.

In 2018, the National Health Commission issued the *Standards for Service Capability of Township Health Centers* [22] and the *Standards for Service Capability of Community Health Service Centers*, establishing the standards for assessing the service capabilities of primary medical and health institutions.

Following that, the National Health Commission issued the *Guidelines for the Evaluation of Service Capability of Township Health Centers* [23] and the *Guidelines for the Evaluation of Service Capability of Community Health Service Centers*, further strengthening the capacity of primary medical services.

In 2022, the *Standards for Service Capability of Health Centers* were updated to reaffirm PHC as a national priority, and to accommodate the challenges and demands of the aging population in China as well as the COVID‐19 pandemic [24]. In the same year, the *Opinions on Promoting the High‐quality Development of Family Doctor Contract Services* was released to expand the coverage of contracted services, gradually forming a system with family doctors as the gatekeepers of health [25].

**Panel 1: PHC system design in China**

**Figure 1:**
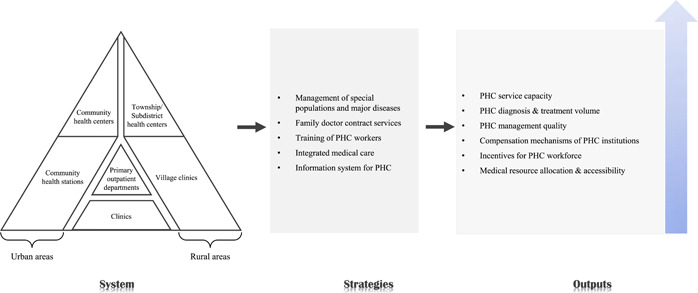
PHC system design in China

Figure 1 illustrates the system design of PHC institutions in China. China's PHC system includes a rural and an urban medical and health service system. Rural areas have better geographical accessibility in townships and villages, while cities take communities as units to achieve full coverage of services within the regional scope. In rural areas, the “tertiary health service network” was designed where county‐level health care institutions (including traditional Chinese medicine hospitals, maternal, and child health hospitals) takes the lead, township health centers shoulder the majority of the workload, and village clinics support the ground‐level services; In urban areas, a medical and health service system based on community hospitals, community health service centers (stations), and clinics has been formed. (PHC, primary health care).

## 3 SERVICE PROVIDER AND COVERAGE

### 3.1 Primary care institutions & professional workforce

#### 3.1.1 Institutions

PHC institutions are the “goalkeeper of public health” in the Chinese health care system, providing generalist clinical care and basic public health service to enhance the universal health coverage (Panel 1). In the past decade, the Chinese government was devoted to establishing a comprehensive network of institutions to promote the accessibility of PHC service (Figure 1). Compared with 2012, the number of PHC institutions in 2021 has increased by 7.1% to 978,000, including 35,000 township health centers and 599,000 village clinics in the rural areas, as well as 10,000 community health centers and 26,000 community health stations in the urban areas. Similarly, the total number of PHC beds increased by 29.3% in the past decade to 1,722,000. Although the PHC system in China is dominated by publicly owned entities, private institutions such as private outpatient departments and clinics are indispensable to the diversity of the PHC provider network. According to the 6th National Health Services Survey, 90% families can reach the nearest health care institutions in 15 minutes [26]. Besides PHC institutions, the 145,000 professional public health institutions (including Centers for Disease Control and Prevention, institutions for health supervision, institutions for maternal and child health care, institutions for health education, etc.) also played significant roles in the Chinese PHC system, providing basic public health service for residents living in both urban and rural areas [13].

In 2015, the Chinese government started to explore the hierarchical diagnosis and treatment system as a capstone of integrated care. The construction of medical alliances is an institutional innovation in the health care reform which allows hospitals and PHC institutions to work closely to provide disease prevention, treatment, and rehabilitation service across the life course. By the end of 2019, China has established 12,000 medical alliances to mobilize high quality health care service from public hospitals to PHC institutions [13].

#### 3.1.2 Workforce

**Figure 2:**
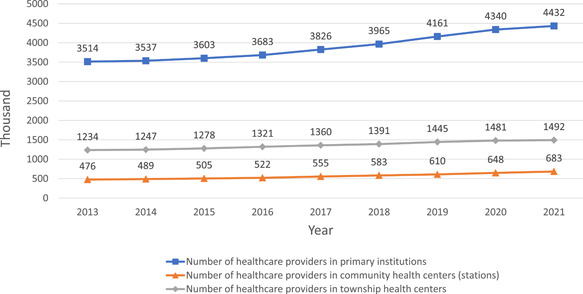
Number of primary health care providers in China, 2013−2021

**Figure 3:**
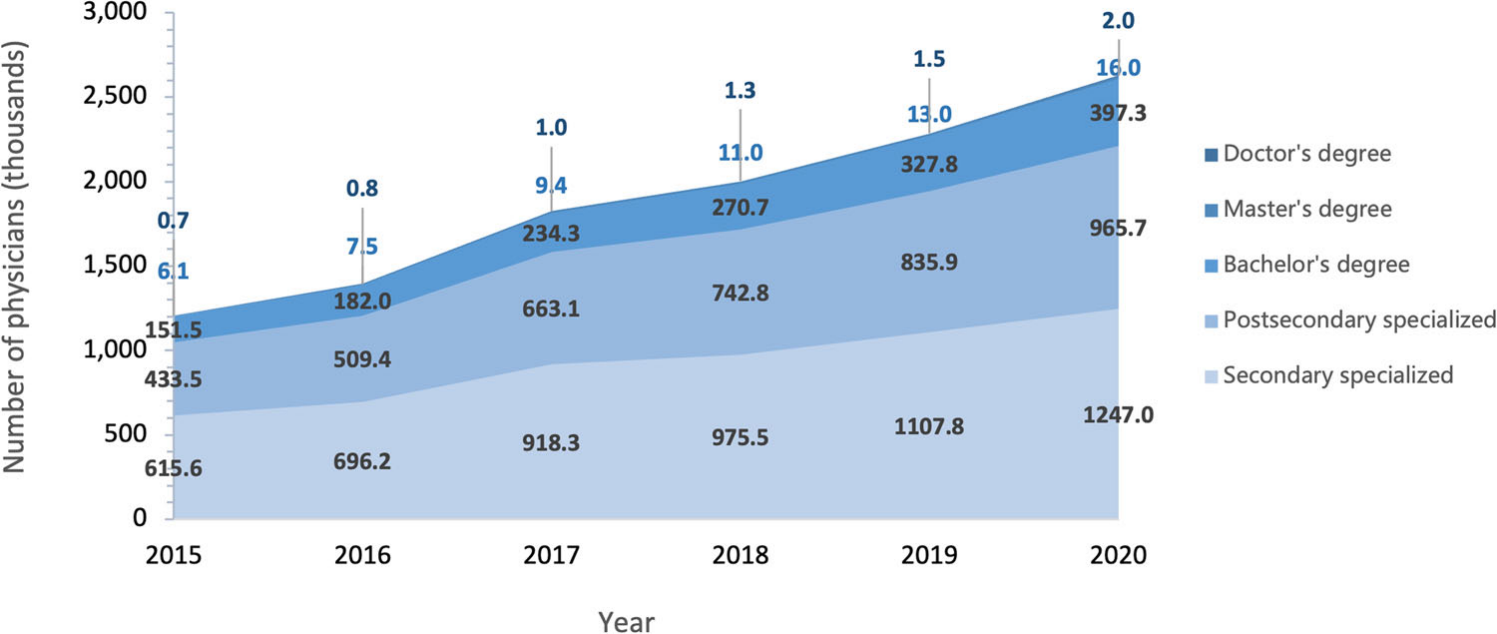
Education level of physicians in primary health care institutions in China, 2015−2020

Adequate supply of qualified health care workforce is the foundation of high‐quality PHC services. General physicians are supposed to be the solution of workforce shortage in PHC reform, the training and qualification of whom have been describe by Li et al. [5] Strategies listed in Panel 2 were proposed to improve the number and quality of health care providers in PHC institutions, especially in rural area, where the health care resources are underserved. PHC personnel in urban and rural areas are generally increasing in the past decade [13]. From 2013 to 2021, the number of PHC providers in China increased from 3,437,000 to 4,432,000 (Figure 2), and registered (associated) physicians increased from 1,009,000 to 1,615,000. In 2020, the average number of physicians and nurses working in each community health care centers were 38 and 6. In rural areas, the total number of doctors has been slightly decreased from 1,255,000 to 1,147,000, while the number of barefoot doctors almost reduced by half, from 1,020,000 to 670,000. However, as the residents have been decreasing in rural areas [9], the doctors per 1000 people has increased from 1.25 in 2012 to 1.3 in 2021. Those promoted to registered doctors or registered associate doctors by formal training can also explain the drop of barefoot doctors, reflecting the quality improvement of PHC workforce as well. However, the distribution of PHC providers' educational background has seen little shift in the past few years. In 2020, more than 75% of the PHC providers had their highest education below bachelor's degree, 13.7% possessed a bachelor's degree, less than 1% are master, while PhD or MD only made up under 0.1% (Figure 3). The distribution indicated that PHC institutions are lack of attraction to labors with higher educational background.

**Panel 2: Professional training**

**Figure 4:**
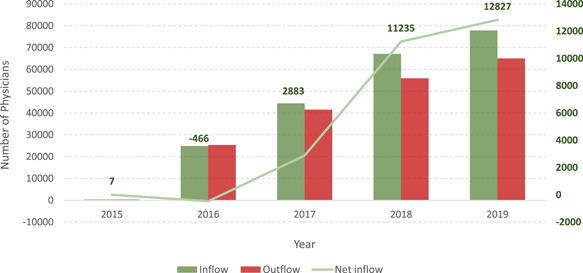
Physician inflow and outflow at primary health care institutions in China

College education, professional education, continuing education, and standardization training of doctors are critical to the sustainable supply of PHC workforce (Figure 4).

a. The *Primary Health Care Personnel Performance Initiative* has trained more than 590,000 PHC providers.
b. The *General Practitioners Training Program* aims to provide training for specialists and rural doctors to become registered general practitioners in PHC institutions. This project has trained 230,000 PHC workers.
c. Free college training has been provided for more than 70,000 rural medical students. More than half of them have been working in rural PHC institutions.
d. “Barefoot Doctors” and “Village Doctors” are two different concepts. Barefoot doctors are not officially registered but were allowed to practice in rural areas in the past. Village doctors include all physicians working in rural areas who have been formally trained and have passed an exam of certification. In the past decade, more than 4300 medical students with bachelor's degrees benefited from the “register without examination policy” and served as “Barefoot Doctors”; 154,000 passed rural associate general practitioner examination.
e. A more reasonable and competitive renumeration system enabled the development of PHC workers. In the past 5 years, more physicians have relocated from hospital to PHC institutions than moving backwards.

### 3.2 PHC services

Unlike hospitals and CDCs, PHC institutions are responsible for both clinical and public health services in China. PHC institutions work closely with professional public health institutions to prevent disease and promote overall health of the residents. According to the hierarchical diagnosis and treatment policy, patients are encouraged to visit and get treatment in PHC institutions first and be referred to hospitals for advanced treatment if needed. Thus, it is critical to promote the capacity and quality of PHC service to satisfy the generalist clinical care requirements of residents.

In 2009, 2011, and 2017, three versions of the *National Public Health Services Program* covering the entire population have been outlined consecutively [18, 21, 27]. The latest list included a total of 55 services available at the primary and the provincial level, falling under the following categories: free provision for urban and rural residents to establish health records; health education; vaccination; health management of children aged 0−6; maternal health management; elderly health management; health management of patients with hypertension and type 2 diabetes, health management of patients with severe mental illness, and health management of patients with tuberculosis; health management by traditional Chinese medicine (TCM); reporting and response to communicable diseases and public health emergencies; and health surveillance, inspection and coordination [21]. These services characterized the equalization of health care in China, an important task in deepening the reform of the medical and health care system, and a signature project that benefits thousands of households and the nation's 1.4 billion population.

**Figure 5:**
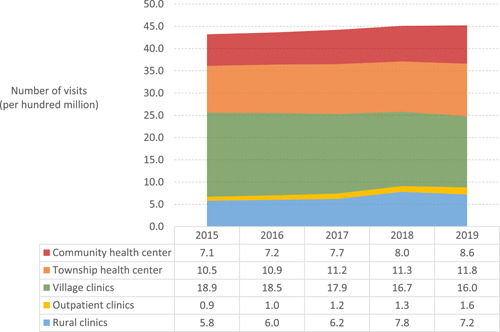
Patient visits per year provided by primary health care institutions in China, 2015−2019

The service capacity of PHC institutions in China has seen great improvement in the past few years. In 2019, the number of hospital beds reached 4.81 per 1000 population in rural PHC institutions, and 1.48 per 1000 population in township health centers [11]. Meanwhile, the number of admissions to PHC institutions was 42.95 million, a decrease of 810,000 compared with 2018 [9, 11]. From 2015 to 2019, the number of patients visited PHC institutions averagely increased 0.6% every year (Figure 5). The visits in outpatient departments increased 12.5% per year, followed by clinics (5.3%), community health care centers (4.5%), and township health centers (2.6%) [11]. Take community health centers (stations) as an example, the total visits in 2019 were 859 million, a 4.17% increase from 2018. Due to the COVID‐19 pandemic, the visits in community health center declined to 755 million in 2020 [28]. Besides generalist clinical service, the public health services in PHC institutions, such as childhood immunization, maternal and child care, elderly health promotion, and chronic disease management, have made great progress as well. In 2020, 127.19 million adults over 65 years of age were reached by health promotion programs; 109.12 million people living with hypertension and 35.73 million people living with type 2 diabetes were routinely managed by PHC institutions.

From 2013 to 2018, the Chinese government took tremendous efforts in the rational allocation of health care resources. In 2018, nearly 90% of residents can reach the nearest health care institution within 15 minutes, a meaningful raise compared with 84.0% in 2013 [13]. The improvement of PHC service accessibility shifted the residents' preference of visit. The 6th National Health Services Survey conducted in 2018 stated that 87.4% of residents would visit county‐level health care institutions for care, 7.4% higher than that in 2013 [26].

### 3.3 Major achievements

#### 3.3.1 Health records

Health records are standardized records created by medical and health institutions as part of the process of providing medical and health services to urban and rural residents. They are file records that follow the residents through the entire life, covering a variety of health‐related information and factors. The establishment of an electronic health record management system and the gradual release of files to residents play an important role in improving the efficiency of various public health services and empowering health management by the residents themselves. In 2019, the filing rate of electronic health records reached 86.82%, and the utilization rate of health records was 55.34% [29, 30].

#### 3.3.2 Health education and promotion

Good health literacy is conducive to changing unhealthy lifestyles and behaviors and improving the overall health of residents. By providing health education materials, creating health educational billboards, conducting public health consultation, organizing health lectures and other activities, primary medical and health institutions in various regions have effectively improved the residents' health literacy. In 2020, the national health literacy level of urban residents was 28.08%, and that of rural residents was 20.02%, up 3.27 and 4.35 percentage points from 2019. Residents in the eastern, central, and western China had health literacy levels of 29.06%, 21.01%, and 16.72%, respectively, an increase of 4.46, 4.70, and 2.42 percentage points over 2019 [31].

#### 3.3.3 National immunization program

In 2011, China implemented the *China Children's Development Outline (2011−2020)*. The immunization planning monitoring and management system covering the national, provincial, municipal, and county levels has also been gradually upgraded, providing comprehensive PHC service to children aged 0−6 years old. In 2020, the coverage of school‐age children in all types of vaccines included in the national immunization program was close to or exceeded 99% [32].

#### 3.3.4 Maternal & child health

The rate of maternal record filing, maternal health management, and postpartum visit rates have generally been increasing over time. The rate of reccard establishment and postpartum visit have remained above 90%; the coverage of maternal management has risen from 80.9% in 2009 to 92.7% in 2020; the neonatal visit rate has always been above 90% [12]. As a result, the indicators of maternal and child health have significantly improved. From 2012 to 2019, the mortality rate of children under the age of five decreased by 69.2%, from 13.2‰ to 7.8‰; the maternal mortality rate per 100,000 people decreased by 27.3%, from 24.5‰ to 17.8‰; and the infant mortality rate decreased by 45.6%, from 10.3‰ to 5.6‰ [11]. Meanwhile, the gaps of these indicators between urban and rural areas have been gradually narrowed in the past decade [11].

#### 3.3.5 Health management of the elderly

The Elderly Health Management Program provides annual health management services for residents aged 65 and over in the jurisdiction, including lifestyle and health assessments, physical examinations, auxiliary examinations, and health guidance. The number of elderly people under health management has increased year by year, reaching an average life expectancy of 78.4 years in 2020 [33].

#### 3.3.6 Chronic disease management

**Figure 6:**
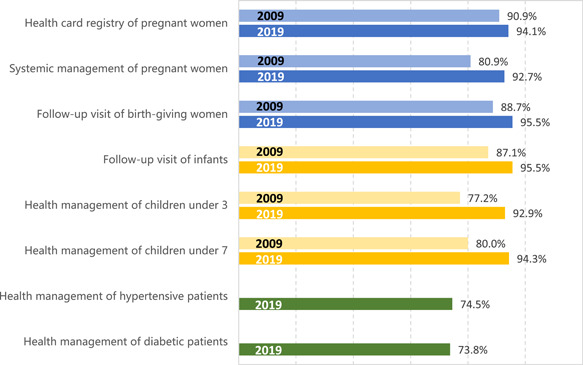
Primary health care coverage of special targeted population, 2009 versus 2019

Chronic diseases are becoming more prevalent among Chinese residents [12], posing greater challenge on chronic disease management by PHC institutions. PHC institutions in China must provide screening, follow‐up assessment, classified intervention based on the patient's condition, and physical examination to patients who have previously been diagnosed with hypertension or diabetes [21]. In 2019, the standardized management rate was 74.54% for hypertensive patients, and 73.76% for diabetic patients (Figure 6). The eastern region had a significantly higher number of chronic disease patients managed than the central and western regions.

According to the National Health Commission's Report on Nutrition and Chronic Disease Status of Chinese Residents [34], chronic disease‐related labor losses have been significantly reduced. The premature mortality rate due to major chronic diseases was 16.5%, a 10.8% decrease from 18.5% in 2015, meeting the 2020 national plan ahead of schedule.

#### 3.3.7 Family doctor contract services

After signing up for family doctor services, residents can obtain basic medical care, preventive health care, and health consultation, among other services. By the end of 2021, family doctor contract services have been promoted across the country, with more than 428,000 teams of family doctor established, and a total of 1.478 million family doctors signed up [28]. The services are primarily intended for chronic disease patients, the elderly, and other targeted population. Long‐term prescriptions, home visit services, and other medical and health services are available.

#### 3.3.8 Traditional Chinese Medicine

TCM health management services primarily covers the elderly over the age of 65 years and children aged 0−36 months. The services include TCM consultation, child TCM diet recuperation, and basic acupoint massage training. Morepatients have sought TCM health management services as PHC institutions became better equipped with TCM functions. TCM outpatient and emergency services in community health service institutions have been steadily rising from 56 million in 2015 to 73 million in 2020 [12].

## 4 FINANCING POLICIES

The lever effect of financial support in quality primary care can be divided into three parts: (1) insurance and payment support for patients in PHC service utilization, (2) subsidies for public health service and (3) financial incentives for PHC provider

### 4.1 Primary medical insurance/payment system

Universal health insurance coverage is a key to the Healthy China Initiative. In 2012, the Chinese government introduced a serious illness insurance system for urban and rural residents and raised the reimbursement rate up to 50% [35]. To narrow the gap between rural and urban areas, the Urban Residents Basic Medical Insurance and Rural New Cooperative Medical Insurance were merged in 2016 [36]. In 2020, the government enacted the *Opinion on deepening reform of the medical security system* to reform medical insurance management, control medical costs, and sustain the balance for medical insurance funds [7]. Diagnosis Related Group or Diagnosis‐Intervention Packet payment of medical insurance were put in place, intended to influence medical behaviors and rightfully compensate doctors. Private insurance companies are also encouraged to participate, producing complementary medical insurance, and serving the diverse demands of the people.

By the end of 2021, the medical insurance has covered 95% of the Chinese population [10]. The per capita subsidies for PHC services reached the new high of 580 CNY. More importantly, the increased reimbursement coverage and rate alleviated the financial burden of disease—over the last few years, 2860 medicines have been included in the medical insurance plan, with 67 prescriptions seeing price reduction over 60% [37]. The poverty assistance reimbursement rates for rural and urban residents have been increased to 70% and 80%, respectively. Outpatient visits for chronic diseases such as hypertension and diabetes were also covered by medical insurance, benefiting around 120 million individuals [26, 38].

There is not enough official guidance in seeking health care services in PHC institution. The lack of awareness of hierarchical diagnostic and treatment practice has made patients with common ailments to prefer hospitals over PHC institutions. Medical insurance, an influential factor for shaping medical behaviors and balancing resource allocation, should be leveraged in future practices.

### 4.2 Public health service subsidies

**Figure 7:**
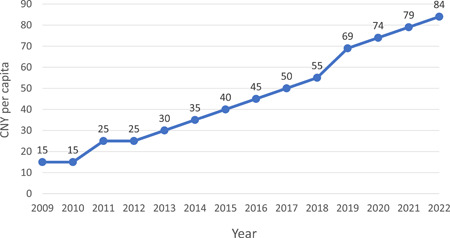
Time trend of government subsidy for basic public health services (CNY per capita)

Supported by national, provincial, and county level government, PHC institutions provide free public health service for residents to prevent disease and promote health. In 2009, the free public health service included 10 items, which was later expanded to 12 in 2017 and 14 in 2021. The government subsidies were budgeted based on the number of people served in the last year and the bill would be settled the following year according to the actual services provided. The subsidies increased from 15 CNY per capita in 2009 to 84 CNY per capita in 2022 [39, 40] (Figure 7). In some developed areas, provincial government allocated more subsidies for public health services than the national average (e.g., 93 CNY per capita in Jiangsu Province). This could effectively motivate the health care providers and ensure the quality of care.

### 4.3 Remuneration system & incentives for quality care

Remuneration system reform has been a continuous work since the 2009 health care reform. The Chinese government has implemented various financial assistance and policies to support PHC workforce. First, the government guaranteed the basic salaries of the staffs in PHC institutions and continued to increase their welfare benefits. Moreover, the “*Two Permissions*” strategy restructured the compensation system of public hospitals—it allows the health care institutions to “deviate from the present wage level of public institutions” and to “use the profit of health care services as an allowance to workers after withdrawing required funding.” This strategy effectively transformed the compensation system for PHC practitioners. Health care service profit generated from the developed areas and government financial subsidies in underdeveloped areas are used to boost the salary of PHC workers.

Aside from financial assistance, the performance evaluation system was rebuilt to improve the satisfaction of PHC workers. In 2015, the *Opinion on Primary Health Care Workers' Professional Titles Evaluation* emphasized that the evaluation should be guided by health care capability, quality, and performance in practice, instead of academic publication record [41]. In 2018, primary physicians who received general residents practitioners training and been awarded intermediate titles were allowed to be awarded with associate senior title after working consecutively in underdeveloped villages for 10 years [42]. Hence, physicians became more willing to work in PHC institutions and provide high quality service.

## 5 TECHNOLOGICAL SUPPORT

The supply of basic medications and health technologies is one of the most essential guarantees for the effectiveness of PHC institutions, and it should be safe, effective, accessible, and affordable. To facilitate the hierarchical diagnosis and treatment system, primary medical, and health care should use information technology to improve the efficiency and inclusiveness of primary medical services, supplemented by management innovations such as specialist alliances and telemedicine collaboration.

### 5.1 Medication

Since the implementation of the essential medicine system in 2009, the Chinese people's medical expenses have been greatly reduced. In the 2 years following 2009, China's essential medicine system was formed, and each province gradually implemented the system in village clinics and extended it to higher‐level hospitals and nongovernment medical institutions. The current National Essential Medicines List [43] includes 685 items (417 western medicines and 268 Chinese patent medicines) that can address patients' clinical needs for common diseases, chronic diseases, and emergency rescue.

China is experiencing a shift in disease spectrum, an aging population, and an expanding population of chronic disease patients. The demand for basic medicine is therefore growing rapidly. At present, China's long‐term medicine prescription management is still in its infancy, and there is no mature paradigm for medicine prescription and management. At the end of November 2021, the National Health Commission issued the *Notice on Promoting the Experience of Graded Diagnosis and Treatment and the Construction of Medical Alliances in Sanming City*, proposing to provide 39 basic medicines for chronic diseases free of charge; improve the interconnection of the catalogues and the inter‐product substitution between institutions; Unify the catalogue of medicines for chronic diseases, establish a long‐term prescription system, and include qualified medical institutions under the coverage of medical insurance.

It is recommended to (1) Strengthen the application of real‐world research in the essential medicine system, the medical security system, and the hierarchical diagnosis and treatment system. (2) Gradually relax the restrictions on medication in PHC settings, so that certain nonessential medicines become available in primary medical and health institutions. (3) Reduce the purchase price of chronic disease medicines through the national centralized procurement. Standardize the settlement of the purchase price of basic medicines with concentrated quantity, cover up the problems such as the long payment collection cycle of the PHC institution and the difficulty of capital turnover of medicine distribution enterprises. (4) Design flexible medicine distribution models, such as the establishment of public medicine warehouses or dedicated distribution companies for remote areas, or the signing of medicine procurement and supply contracts and quality assurance agreements with distribution companies.

**Panel 3: Major challenges of medication use in primary care settings**

a. Listing and supply

The medicine inventory of primary medical and health institutions is restricted, and there is a shortage of doctors and medications at the township level. Patients are often forced to travel to large hospitals in cities for medical services and prescriptions, further weakening township medical care's diagnosis and treatment skills and creating a vicious cycle.

b. Referral and prescription

The existing spectrum of medicines in PHC institutions does not adequately cover the diversity of medicines required by patients seeking referral. PHC institutions have limited inventory space and the number of pharmacists, making replenishment difficult. At the same time, medicine catalogues from different levels of medical institutions have not been linked. Large hospitals lack the motivation to encourage patients to return to local PHC institutions. Under such situation, the long‐term prescription system requires patients to travel back and forth many times; however, if the medication is taken all at once, it is likely for it to be wasted or inappropriately administered in combination. A better mechanism for stable long‐term prescription within the interconnected PHC network is urgently warranted.

c. Delivery and cost

Due to high storage and delivery cost associated with remote locations and extreme traffic conditions, the pharmacy manufacturing companies and the distribution companies have limited incentives to distribute medicines to remote places. Moreover, due to its sole reliance on the medical insurance fund, the actual reimbursement level for national essential medicines has been lower than that specified by the policies.

### 5.2 Health technology application

In 1991, the Chinese government has proposed a 10‐year national plan to select and promote 10 health technological achievements each year [44]. In 2004, the government funding to support “*The pilot program of rural health technology promotion*” was launched in 10 provinces, municipalities, and autonomous regions. In 2008, the project was expanded to 17 provinces. According to the 2009 *Opinions of the State Council on Deepening the Reform of the Medical and Health Care System*, PHC institutions need to define the scope of services, clarify the standards and norms for the use of appropriate technologies, equipment and medicine, and provide public welfare services for the general public with low‐cost and high‐quality [7]. In fact, only 60−80 out of the total of 1500 catalogued technologies are commonly used in PHC settings [45].

The main problems faced by health technology in PHC settings include the following: the existing indicators are relatively outdated, which is not conducive to the access and management of health technology. Insufficient funding and incentives, limited training methods and technical promotion channels, and lack of long‐term effective financial support are also limiting factors for technology application in PHC in China.

In this regard, it is recommended that the government (1) provide policy support and subsidies for the promotion of technological devices; (2) encourage information sharing among medical and health institutions at all levels and promote hierarchical diagnosis and treatment policies. If a problem is found in the application process of a certain technology in PHC settings, regional medical alliances  can be of guidance in a timely manner; (3) establish the knowledge base of health technology, which summarizes, analyzes, and compares the performance and quality of health technologies at the PHC institutions; (4) regularly subsidize free training for professional and technical personnel to study in more advanced provinces and cities, and invite experts to teach technology pertaining to common diseases in the remote areas.

### 5.3 Information system in PHC settings

Remote diagnosis and Internet medical care are also conducive to the promotion of appropriate technologies, such as policy release, information disclosure, technical inquiry, and project management. At present, the health technology promotion platforms in Zhejiang, Sichuan, Shanxi, Hebei, and Anhui are relatively complete in function. The establishment of the operation and management mode of telemedicine information system has improved the level of primary medical management. The orderly, stable, and sustainable operation will continue to provide technical support for rural residents and PHC medical personnel to share high‐quality medical resources.

The National Health Informatization Survey Report released in 2021 investigated the construction of the information system of PHC institutions such as community health service centers, community health service stations, township health centers, and village clinics. The survey found that in the Estern region, community health service centers/stations and township health centers had the highest coverage rate of information system, reaching more than 86%. The construction of the information system in village clinics took the lead in the central region, with a coverage rate of nearly 80%, but the overall information system coverage of the community health service centers/stations is low. The number of PHC institutions in the western region is relatively small, but the coverage of information systems is on a reasonable level (ranging from 67.0% in community health stations to 86.8% in township health centers) [46].

## 6 CHALLENGES AND PROSPECTS

Ever since the Alma Ata Declaration, the scope of health promotion was expanded from merely the responsibility of hospitals and doctors to a wider range of community participation, social involvement, and human rights and equity. The major challenges of PHC in China stemmed from the rapid economic and social transition, where the aging population and growing burden of chronic diseases put forward higher demands for the health care system. To meet the demand of better, efficient, and accessible health care services, guaranteeing basic services and strengthening PHC are the effective solutions. The past decade has witnessed the critical steps of PHC development in China, but the current system remains suboptimal in its capacity of services, quality of delivery, and affordability of care.

As a member nation of WHO, China issued the blueprint for health care development *Healthy China 2030* in 2016, in which the Healthy China initiative has been incorporated into the national strategic plan to ensure the sustainable development goal. It is prime time to transform the blueprint into actions, particularly in the areas of implementing policies at the ground level, optimizing the structure of PHC personnel, and ensuring adequate financial and technological support. More importantly, based on the abundant survey and administrative data, evidence‐based methods should be leveraged to evaluate the implementation process, preferably in a complex intervention framework, to identify problems and set priorities on a scientific basis.

## AUTHOR CONTRIBUTIONS

**You Wu**: conceptualization (supporting); data curation (supporting); visualization (lead); writing − original draft (equal). **Zeyu Zhang**: data curation (supporting); investigation (supporting); writing − original draft (equal). **Ning Zhao**: data curation (supporting); project administration (lead); writing − review & editing (supporting). **Yue Yan**: investigation (equal); visualization (supporting). **Lina Zhao**: investigation (equal); visualization (supporting); writing − review & editing (supporting). **Qi Song**: investigation (equal); writing − review & editing (supporting). **Rong Ma**: investigation (equal); writing − review & editing (supporting). **Changfeng Li**: investigation (equal); writing − review & editing (supporting). **Jinyi Li**: investigation (equal); writing − review & editing (supporting). **Suibin Liu**: investigation (equal); writing − review & editing (supporting). **Xinran Bi**: investigation (equal); writing − review & editing (supporting). **Zongjiu Zhang**: conceptualization (lead); project administration (equal); resources (lead); supervision (lead); writing − review & editing (equal).

## ACKNOWLEDGEMENT

None.

## CONFLICT OF INTEREST

The authors declare no conflict of interest.

## ETHICS STATEMENT

No human subject was directly involved in the present study.

## INFORMED CONSENT

None.

## Data Availability

The data that support the findings of this study are available on request from the corresponding author. The data are not publicly available due to privacy or ethical restrictions.
